# Be coherent and become heard: The multidimensional impact of narrative coherence on listeners’ social responses

**DOI:** 10.3758/s13421-020-01092-8

**Published:** 2020-09-08

**Authors:** Lauranne Vanaken, Dirk Hermans

**Affiliations:** Centre for the Psychology of Learning and Experimental Psychopathology, Faculty of Psychology and Educational Sciences, KU Leuven, Leuven, Belgium

**Keywords:** Narrative coherence, Social function of autobiographical memory, Social relationships, Capitalization of positive emotion, Psychological well-being

## Abstract

Previous research has suggested that sharing autobiographical memories in a coherent manner has a beneficial impact on consequent social reactions of listeners. In this experimental study, we were able to replicate earlier findings by demonstrating that listeners (*N* = 107) showed significantly more willingness to interact with, more social support towards, and a more positive attitude towards coherent than incoherent narrators. Remarkably, these beneficial effects of coherence were observed only for narratives about positive memories. Results are explained in the light of the relevance of positive memories for the social bonding function of autobiographical memory. Furthermore, earlier work was extended and refined by investigating effects of the individual constituting dimensions of coherence (context, chronology, theme) on social responses. In line with our predictions, the dimensions of chronology and theme were most important in impacting social responses of listeners. Possibly a reduction of the attraction effect due to increased effortful processing and reduced credibility due to insufficient emotional elaboration might explain these results respectively. Furthermore, social responses were worse when narratives were incoherent with regard to more than one dimension, in line with the expected additive effect. Overall, fully incoherent narratives, which had had low scores on context, chronology, and theme, had the most adverse effect on listeners’ social responses. This study adds significantly to the domain of memory and cognition by showing how cognitive psychological research would benefit from extending a merely intrapersonal perspective to include an interpersonal perspective that considers social implications of memory and cognition as well.

## Introduction

### The social function of autobiographical memory

The autobiographical memory is responsible for the recollection of past experiences that are personally meaningful and for connecting them to narratives (Fivush, 2011; Tulving, 1972, 2002). Remarkably, the experiences that make up the autobiographical memory are very likely to be retrieved and narrated to others soon after experiencing them (Rimé, 2009; Rimé, Finkenauer, Luminet, Zech, & Philippot, 1998; Rimé, Mesquita, Philippot, & Boca, 1991). The process of remembering in the presence of others, even if no interaction between listener and teller occurs, is referred to as *collaborative remembering* (Meade, Harris, Van Bergen, Sutton, & Barnier, 2018; Thompson, 2008). Not only does collaborative remembering occur very frequently, it serves a crucial function. By reminiscing about our past, we can create, maintain, and enhance social relationships, which is referred to in the literature as the social or the self-in-relation function of autobiographical memory (Bluck, 2003; Bluck & Alea, 2002; Bluck, Alea, Habermas, & Rubin, 2005; Fivush, Haden, & Reese, 1996; Hyman & Faries, 1992).

### Narrative coherence in relation to social functioning

Phenomenological research has identified certain characteristics of autobiographical narratives that are able to impact the extent to which memory’s functions are successfully fulfilled (Alea & Bluck, 2003). One important characteristic that has been studied in this regard is narrative coherence (Vanaken, Bijttebier, & Hermans, 2020). For several decades now, narrative coherence has been deemed the fundamental cornerstone for communication (Labov, 1970). Coherence has since become a topic of research in multiple domains, being defined in slightly different ways across domains (e.g., Adler, Waters, Poh, & Seitz, 2018; Baerger & McAdams, 1999; Habermas & Bluck, 2000; Lysaker & Lysaker, 2002; Reese et al., 2011). In our study, we refer to coherence as a cognitive skill needed to structure single-event narratives, also coined *local coherence* (Reese et al., 2011). This is dissimilar to global coherence, in which full-life narratives are investigated instead of single high-impact events (Baerger & McAdams, 1999). In Reese’s cognitive approach (2011), a narrative of an autobiographical experience is described as coherent when “it makes sense to a naïve listener – not just in terms of understanding when, where, and what event took place – but also with respect to understanding the meaning of that event to the narrator” (Reese et al., 2011, p. 425). To measure the coherence of narratives, a coding system was developed, called the “Narrative Coherence Coding Scheme” (NaCCS) (Reese et al., 2011). The system evaluates narratives based on three dimensions: context, chronology, and theme, which are all scored individually (0–3) and add up to a score of total narrative coherence (0–9). Context refers to whether the narrator indicates the specific time and place of the described events. The dimension of chronology is evaluated on the logical and chronological order of the narrated events. Theme is scored according to the personal interpretations and emotional elaborations the narrator makes, whether he/she can connect the event to other events or to the self, derive meaning from what happened, reach closure, and come to a resolution.

### Correlational evidence

Interestingly, narrative coherence has been shown to be related to interpersonal and social outcomes. For instance, in a study of Waters and Fivush (2015), narrative coherence was positively related to having positive social relationships, measured as a combination of perceived social support, social well-being, and generativity. Moreover, in a study of Burnell, Coleman, and Hunt (2010), veterans with a coherent narrative about past emotional events perceived communication with family to be more pleasant, and they experienced societal opinions to be more positive. In contrast, veterans with incoherent narratives found communication unsatisfactory, feeling prevented from talking about their war memories, because of perceiving both their social circle of family and friends as well members of society to be less interested and misunderstanding. Relatedly, Oppenheim, Wamboldt, Gavin, Renouf, and Emde (1996) observed that couples whose co-constructed narratives of their child’s birth were more coherent, had higher concurrent and longitudinal marital satisfaction. Summarized, it is suggested that sharing narratives in a coherent manner is related to having a higher quality of social relationships.

### Experimental evidence

As illustrated above, research has so far been mainly correlational in nature (Burnell et al., 2010; Oppenheim et al., 1996; Waters & Fivush, 2015). The existing experimental work has chiefly focused unidirectionally on the impact of the social context on memories and the way we talk about them (Grysman & Mansfield, 2017; Pasupathi, Stallworth, & Murdoch, 1998; Pasupathi, 2001; Pasupathi & Rich, 2005). For instance, in a study of Bavelas, Coates, and Johnson (2000), distracted listeners, as opposed to attentive listeners, caused the narrator’s performance to decline, resulting in a narrative that was poorer in structure.

However, research that concerns the opposite direction of the relationship, i.e. that investigates the impact of narrative coherence on the social context, is lacking. Nonetheless, it is important to study memory qualities that can enhance or disturb the social bonding function of autobiographical memory, since these qualities are subsequently able to impact our social relationships (Vanaken et al., 2020), which in turn have been shown to be of crucial importance to our psychological well-being (Baumeister & Leary, 1995; Harandi, Taghinasab, & Nayeri, 2017).

In this regard, we set up the first experimental study in the domain to examine if coherent narrators, as compared to incoherent narrators, can evoke more positive social responses in listeners (Vanaken et al., 2020). Using a within-subject experimental design, participants listened to four pre-recorded audio clips in which the speaker narrated a positive or negative autobiographical experience in either a very coherent or a very incoherent manner. After each audio fragment, participants (i.e., the listeners) are asked to indicate their social reactions towards the narrator by filling out questionnaires. Results were in line with our predictions: listeners showed more willingness to interact, more instrumental support, more positive feelings, more empathy, and more trust towards those narrators who talked in a coherent manner about their autobiographical memories, as compared to those who talked in an incoherent manner. Importantly, negative feelings in the listener were evoked when the speaker talked incoherently, but especially when it concerned a positive memory (Vanaken et al., 2020).

### Present study

#### First aim: Replication

Since the former study (Vanaken et al., 2020) was the first to empirically support the impact of narrative coherence on listeners’ social responses, replication is needed. Keeping in mind that science is facing a replication crisis (e.g., Diener & Biswas-Diener, 2020; Moonesinghe, Khoury, & Janssens, 2007; Pashler & Wagenmakers, 2012), it is important to rule out possible false-positive effects of initial studies. Hence, the *first* aim of the current experiment was to investigate whether the previously observed effects are replicable and thereby examine if narrative coherence is more positively socially reacted upon than narrative incoherence. We predicted, in line with the results from our previous study, that coherent narrators will be more positively socially evaluated by listeners, as compared to incoherent narrators.

#### Second aim: Extensions

##### Part A: The multidimensional investigation of narrative coherence

In the present study, the *second* aim concerns building upon previous findings by refining and extending our work in two ways. First, in Vanaken et al. (2020), we used the typical all-or-nothing approach for experimental work, in order to investigate if there was an effect of narrative coherence on social responses in the first place. This means that coherent narratives scored high on all three dimensions – context, chronology, and theme (Coherent: CON = 3, CHR = 3, THE = 3). Conversely, incoherent narratives were completely incoherent in terms of contextual, chronological, and thematic coherence (Incoherent: CON = 0, CHR = 0, THE = 0). However, in this study, we wanted to refine the approach and look deeper into the effect of the individual constituting dimensions of coherence (context, chronology, theme), since it is essential to know which dimension(s) is/are crucial in affecting listeners’ social responses. Hence, in the present study, we did not only use fully coherent and fully incoherent stories, but also stories that can be situated in between, by manipulating the coherence of each individual dimension that constitutes total narrative coherence (Reese et al., 2011). To ensure a strict and clear multidimensional manipulation, we used the extreme scores on each of the three dimensions (score 0 vs. score 3), resulting in eight (2^3^) different versions of each narrative. As a result, we were able to not only investigate the effect of context, chronology, or theme on social responses, but also all their possible combinations, whilst keeping the story topic constant over the different manipulations of coherence. We predicted that stories scoring lower on coherence will lead to more negative social responses, in particular when the lacking dimensions concern chronology or theme. We expected the effect of a missing context to be smaller in impacting the social responses. These predictions are based on our own previous work on the cross-sectional and longitudinal relations between narrative coherence and social bonding, from which we know that associations with social functioning are the strongest for chronological and thematic coherence (Vanaken & Hermans, 2020). Individuals who were more chronologically coherent, experienced fewer negative social interactions – at the same moment in time, but also 5 months later. Conversely, individuals who experienced less negative social interactions were more chronologically and thematically coherent 5 months later. The quality of close relationships also contributed significantly to better narrative coherence, and in particular to better thematic coherence at follow-up. Relatedly, the multiple dimensions of coherence are suggested to be differentially related to various outcome measures (e.g., some are more important for identity, others more for communication, etc.) (Reese et al., 2011). Moreover, we predicted an additive effect of the individual dimensions, meaning that the more incoherent the narrative becomes, or in other words the larger the number of dimensions that receive a low score, the worse the social response becomes. This prediction stems from literature showing both theoretical and empirical evidence ﻿that supports the view that the dimensions that narrative coherence consists of are largely independent (Reese et al., 2011). The dimensions are suggested to reflect skills that develop over time, and are a unique, independent contribution to the concept of narrative coherence as a whole (Reese et al., 2011). Therefore, we expected them to add up in their effect on social reactions, rather than interact with each other or multiply the effect.

##### Part B: The multidimensional investigation in positive and negative memories

Second, in our previous experimental study (Vanaken et al., 2020), we already used high-point and low-point memories, since the literature suggested that coherence is traditionally investigated in personally meaningful positive (high points) and negative experiences (low points) (McLean, Pasupathi, Greenhoot, & Fivush, 2017; Reese et al., 2011). However, the extension in the current study concerns the endorsement of the previously described in-depth multidimensional approach in these high-point memories as well as in the low-point memories. Hence, in this experiment, participants listened to 16 narratives, eight positive ones and eight negative ones, all differing in their degree of coherence (per valence) and administered in a randomized order, with the restriction of having no more than two consecutive narratives of the same valence. We improved on our previously used procedure (Vanaken et al., 2020) by keeping the story topic constant, over the different (in)coherent versions of the story. Coherence of positive and negative stories were investigated separately, expecting that, in particular in narratives about positive experiences, the more incoherent the story is, specifically with regard to theme and chronology, the more negative the social responses will be. This prediction is based on results of our previous study (Vanaken et al., 2020), in which we observed that negative feelings were evoked when the speaker talked incoherently, but especially when it concerned a positive memory. Also, the prediction is based on the idea that positive autobiographical memories are, in comparison to negative ones, more frequently used to bond with other people. For instance, McLean and Lilgendahl (2008) showed that high points are more frequently endorsed for social functions, like informing others, or as topics of conversation, in comparison to low points. Moreover, Alea, Arnaud, and Ali (2013) let participants write about events that served a self, social, or directive function, after which they asked them to report the memory’s valence. They found that, across different age groups, memories used for social functions were the most positive, whereas directive memories were the most negative. Rasmussen and Berntsen (2009) had very similar results in a study with a comparable design, also indicating that social memories were dominated by positive emotion. Therefore, it is suggested that coherence would be of greater importance in impacting social reactions when narratives concern positive memories, since these are adopted more frequently for social bonding purposes.

#### Predictions

In summary, in the current experiment, we used a procedure that is similar to Vanaken et al. (2020), although extended and refined, in order to investigate whether narrative coherence is more positively socially responded to than incoherence, and if so, which dimensions of coherence are crucial in impacting those social responses, and for which valence these differences might matter the most. In a within-subject experimental study, participants listened to 16 pre-recorded audio clips in which the speaker narrates a positive or negative autobiographical experience in a manner that varies with regard to each dimension of coherence. After each audio fragment, participants are asked to socially respond to the narrator, by filling out questionnaires. We decided a priori upon a small selection of social response measures for our study, since they have proven to be of importance based on our own previous results and the work of others (Burnell et al., 2010, Coyne, 1976; Vanaken et al., 2020; Waters & Fivush, 2015). We chose to measure listeners’ willingness to interact with the narrator, their social support (emotional and instrumental) towards the narrator, and their positive and negative attitude towards the narrator. We predicted, in line with the results from our previous study, that listeners will respond more positively towards coherent narrators on the aforementioned social outcomes, in comparison with towards incoherent narrators. Furthermore, we predicted that especially chronology and theme are of importance in affecting the social responses. We expected an additive effect of the individual dimensions, indicating that the absence of a combination (i.e., more than one) of dimensions will worsen the social response more than the absence of a single dimension, in particular when the shortcoming dimensions concern chronology or theme. We predicted to see given effects of coherence especially in positive narratives, more so than in negative narratives. In other words, we expected that listeners will be more willing to interact with, more supportive towards, and have a less negative and more positive attitude towards those people who narrate coherently about their positive autobiographical memories, particularly with regard to thematic and chronological coherence, in comparison to towards incoherent narrators.

## Methods

### Participants

A total of 107 participants between the ages of 18 and 33 years (*M* = 19.57, *SD* = 2.51) participated in the study, of which 95 (88.8%) were female and 12 (11.2%) were male. Sample size was determined before any data analysis, based on previous similar work (Vanaken et al., 2020). Our sample in the current study was very homogeneous, consisting of mostly young white female students, with only four participants older than 25 years. All of them were Belgian and indicated Dutch as their mother tongue or indicated actively speaking it. Participants signed up via the Experiment Management System (EMS) of the KU Leuven. All gave written informed consent before the start of the study and received either one course credit or remuneration (€8) for their participation. The study was approved by the KU Leuven Social and Societal Ethics Committee (G - 2018 03 1175). Note that we pre-registered the study with the aim of collecting a minimum of 100 participants, however, we were able to secure data from seven extra participants (https://aspredicted.org/blind.php?x=k7jj8r).

### Material and measures

#### Narratives

Since our study partially aimed to replicate our own previous work, materials and measures are an adapted version of Vanaken et al. (2020). In this study, we report all measures, manipulations, and exclusions. We created 128 narratives (in Dutch) based on themes that are very common in this sample and representative for self-reported events with high emotional impact (McLean & Breen, 2009). We wrote the narratives based on our extensive experience in collecting and coding hundreds of narratives in similar samples (e.g., Vanaken & Hermans, 2020). We used eight positive (travelling, birthday party, falling in love, graduating, gap year, moving house, birth of sibling, music festival) and eight negative topics (divorce of parents, passing away of grandma, suicide of friend, break-up of relationship, end of friendship, cancer diagnosis of mother, mental health issues, failing exams). For each of the 16 topics, we wrote eight unique narratives, differing systematically in their degree of coherence according to the Narrative Coherence Coding Scheme, to ensure strict manipulation of the different dimensions (Reese et al., 2011). An example of eight narratives on the same topic, differing in their degree of coherence is shown in Appendix 1. All 128 narratives that were used in the study are included in the Open Science Framework page of this study (10.17605/OSF.IO/3CTXV ).

The Narrative Coherence Coding Scheme (Reese et al., 2011) entails three separate dimensions (score 0–3) that are summed up to reach a total narrative coherence (score 0–9). The first dimension is “context,” which refers to how the narrator orients the event in time and space. If the narrator does not provide any information about time or place, score 0 is assigned. If there is partial information, meaning that only the time in which or the location where the event took place are mentioned, at any level of specificity, a score of 1 is assigned (specific time, e.g.: when I was 7 years old; nonspecific time, e.g.: when I was a child; nonspecific place, e.g.: when I was abroad; specific place, e.g.: at my grandmother’s house). A score of 2 is assigned when both time and location are provided, but no more than one dimension is specific. When time as well as location are mentioned both specifically, a score of 3 is given. The second dimension is “chronology,” which refers to whether the narrator describes the components of the events along a (chrono)logical timeline. If the narrator describes less than three actions of which the total event consisted (very short narratives like: when my mother passed away), a score of 0 is assigned. If the narrator describes more than three actions but fewer than half can be ordered on a timeline by a naïve listener, a score of 1 is given. When more than half of the actions can be ordered on a timeline by a naïve listener, a score of 2 is assigned. A score of 3 is given when almost all actions can be ordered on a timeline and the narrator uses words (e.g., right before, after an hour, the next day) to mark the temporal order of the actions. The third dimension is “theme,” which refers to whether the narrator can maintain and elaborate emotionally on a topic, if he/she can come to a resolution or is able to reach closure. Score 0 is given for narratives that are substantially off topic or are difficult to be defined in terms of a certain theme (possible themes could be, e.g., the loss of a family member, a car accident, marriage). If the topic is identifiable, but minimally elaborated upon with personal evaluations (e.g., I felt really sad, I was full of joy), a score of 1 is assigned. Score 2 is assigned when narratives are substantially developed around a theme and there are multiple personal interpretations or evaluations given. Finally, a score of 3 means that the narrator can take some meta-perspective on the event, and doesn’t only elaborate on it with momentary feelings or evaluations, but can also link it with other autobiographical events (e.g., that reminded me of the first time I fell in love), or can come to a resolution (e.g., that event made me realize how important family is for me) or reaches closure (e.g., I feel like in the end I was able to give the event a place and move on with life).

To create different versions of the narrative, we used the extreme scores (score 0 vs. score 3) on each of the three dimensions of coherence (context: CON, chronology: CHR, theme: THE). Thereby, we created eight (=2^3^) versions differing in their degree of coherence. This approach allowed us to also investigate every possible combination of the dimensions (e.g., high on context and high on chronology, but low on theme). Narratives were thus coded in the following way: CON CHR THE (CON = 3, CHR = 3, THE = 3); No CON (CON = 0, CHR = 3, THE = 3); No CHR (CON = 3, CHR = 0, THE = 3); No THE (CON = 3, CHR = 3, THE = 0); No CON No CHR (CON = 0, CHR = 0, THE = 3); No CHR No THE (CON = 3, CHR = 0, THE = 0); No CON No THE (CON = 0, CHR = 3, THE = 0); No CON No CHR No THE (CON = 0, CHR = 0, THE = 0).

Subsequently, three colleagues with expertise in the field independently coded the 128 narratives for coherence using the Narrative Coherence Coding Scheme (NCCS), whilst blind for conditions. Perfect inter-rater reliability (100%) was established, indicating that we used very clear examples of coherence versus incoherence.

Afterwards, 16 different individuals, all women aged between 22 and 28 years, voice-recorded the eight versions of one topic, so no voice was exclusively linked to a certain level of coherence. All stories had a word count of between 195 and 263 words, resulting in a spoken duration of between 63 and 91 s. We used audio clips instead of video clips to reduce possible effects of race, attraction, or social preference. Furthermore, audio clips were chosen instead of narratives that participants would have to read, to increase the chances that participants would not skip over large parts of text that they were supposed to read and, predominantly, to increase the ecological validity of this study. In a social context, individuals narrate verbally about their experiences and listen to each other, instead of writing them down and having them read by each other. The choice for female voices was made because of a better match between speaker and listener (88.8% female) characteristics (Alea & Bluck, 2003). Higher similarity between speaker and listener stimulates conversations to become more in-depth and to evoke more personal reactions and emotions, which is important since we were interested in exactly these individual differences in personal reactions towards (in)coherent narrators (Alea & Bluck, 2003).

#### Social responses

We used questionnaires to investigate listeners’ social responses with respect to the (in)coherent stories. We decided a priori to focus on three types of social responses, based on previous research (Vanaken et al., 2020), which were willingness to interact, social support (emotional and instrumental), and attitude (positive and negative), all measured on a 6-point Likert scale.

We measured willingness to interact with the speaker, using a questionnaire of Coyne (1976). The questionnaire consisted of eight questions, each to be answered on a 6-point Likert scale (ranging from “Definitely not” to “Definitely yes”), giving a possible minimum score of 8 and a maximum score of 48. Questions contained, for example, the willingness to meet the other, seek advice from the other, and sit on the bus with the other.

We measured social support with the 2-Way Social Support Scale of Shakespeare-Finch and Obst (2011). We used three items measuring emotional support and three items measuring instrumental support. Emotional support assessed elements like: “I would be there to listen to his/her problems.” Instrumental support included items such as: “I would help him/her when he/she is too busy to get everything done.” Both were rated on the same 6-point Likert scale (ranging from “Definitely not” to “Definitely yes”), resulting in a minimum score of 3 and a maximum score of 18 for emotional support, as well as for instrumental support. ﻿Cronbach’s alpha coefficients point to a moderate to high internal consistency of the subscales (ranging from .81 to .86 in different samples) (Shakespeare-Finch & Obst, 2011).

We assessed positive and negative attitudes towards the narrator using one item each (To what extent do you have a positive attitude towards the other? To what extent do you have a negative attitude towards the other?). Each question was again to be rated on a similar 6-point Likert scale (ranging from “Not at all” to “Very much”), giving a minimum score of 1 and a maximum score of 6 on each of the two items.

#### Procedure

Procedural elements were also similar to our previous study (Vanaken et al., 2020). Participants were invited to the lab in groups of a maximum of six people and were first given general information about the aim of the study. They were told that that they would be participating in a study on memory processes and aspects of social-psychological functioning. Then, the participants were asked to take place in an individual cubicle (soundproof cabinet in which they sat behind a table facing only the computer, which was connected to headphones) and to carefully read the informed consent. Herein, we stated, along with all the necessary ethical information, that we were trying to obtain insight into how individuals react when listening to memories of other people. Upon agreement, they were informed that they would hear 16 different people talking about a personal memory. They were asked to pay close attention to each audio clip, as further questions about them would follow. They were made aware that they could withdraw from participation at any time. Then, if the participants did not have any further questions, the headphones were put on and the experiment was initiated.

In the experiment, the narratives were administered in randomized order (computer-based randomization), with the restriction that there would be no two subsequent stories of the same valence or of the same level of coherence. Each participant hence received eight positive and eight negative stories, each with different levels of narrative coherence. Consequently, the participant listened to 16 different stories in a random order, with each story narrated by a different voice. Each of the 16 audio clips was followed by questions to assess participants’ social responses towards the narrator. The questions regarded willingness to interact, social support (emotional and instrumental), and attitude (positive and negative). When participants finished the experiment, they were thanked for their participation and given a debriefing letter to take home, including contact details of the researchers as well as instances for mental support, in case of any further questions or difficulties after their participation.

## Results

### Analyses

Analyses were executed using IBM SPSS Statistics 26 and were in line with our pre-registration. Our *first* question concerned the replication of the earlier found beneficial effect of total narrative coherence (in comparison to total incoherence) on social responses. Therefore, we conducted a repeated-measures (RM) MANOVA, in which we compared the general social response score (sum score of different dependent variables) for the two extreme versions of coherence (fully coherent vs. fully incoherent). Afterwards, this was followed up by RM ANOVAs per dependent variable, to examine if different social reactions were possibly differently impacted by the total level of narrative coherence.

With regards to the *second* question, we aimed to refine the previously observed results by investigating the impact of each individual dimension of coherence on social responses, as well as their respective combinations. Therefore, we first ran a RM MANOVA, in which we compared the social response score for the eight different levels of coherence. Subsequently, we conducted a 2 × 2 × 2 (CON vs. No CON × CHR vs. No CHR × THE vs. No THE) RM MANOVA to investigate which dimension(s) of coherence would be significant in impacting the general social response. Then, a 2 × 2 × 2 (CON vs. No CON × CHR vs. No CHR × THE vs. No THE) RM ANOVA was run for each dependent variable separately to investigate effects of the dimensions on the different social responses specifically.

An alpha level of .05 was set for all analyses. Variables were analyzed using the mean scores on all questionnaires, ranging from 1 to 6. Since the tradition on narrative coherence investigates both low points as well as high points, and since we hypothesized seeing the effects of coherence in positive narratives in particular, we analyzed social responses of negative and positive narratives separately.

### Descriptive results

Descriptive statistics for each dependent variable, being willingness to interact, emotional support, instrumental support, negative attitude, and positive attitude are presented in Table 1. Note that the mean scores are reported for each variable. For instance, the difference between the two extremes (4.79 and 4.42) for the WIL scale indicates that for the eight items on this scale, an average of three items received a rating 1 point higher on the 6-point Likert scale in the “CON CHR THE condition,” compared to the “No CON No CHR No THE condition.”

**Table 1:** Descriptive statistics for social responses

	Willingness to interact		Emotional support		Instrumental support		Negative attitude		Positive attitude	
	*M*	*SD*	*M*	*SD*	*M*	*SD*	*M*	*SD*	*M*	*SD*
*Negative*										
CON CHR THE	4.42	0.94	5.03	0.93	3.81	1.07	1.78	1.11	4.56	0.95
No CON	4.45	0.89	5.01	0.87	3.87	1.06	1.80	1.12	4.53	0.96
No CHR	4.40	0.90	5.09	0.93	3.88	1.02	1.81	1.11	4.64	0.88
No THE	4.49	0.82	5.08	0.88	3.89	1.10	1.69	0.91	4.68	0.95
No CON No CHR	4.36	0.96	4.94	1.00	3.77	1.08	1.81	1.04	4.58	0.98
No CHR No THE	4.46	0.82	4.98	0.93	3.92	0.99	1.83	1.09	4.50	1.08
No CON No THE	4.41	0.86	5.06	0.81	3.98	1.01	1.79	0.91	4.77	0.92
No CON No CHR No THE	4.42	0.89	5.03	0.95	3.86	1.02	1.75	1.06	4.62	0.97
*Positive*										
CON CHR THE	4.79	0.78	4.97	0.80	3.85	0.99	1.53	0.78	5.00	0.80
No CON	4.67	0.84	4.83	0.91	3.77	1.02	1.68	0.97	4.86	0.85
No CHR	4.63	0.91	4.75	0.93	3.65	1.08	1.65	1.06	4.77	0.91
No THE	4.60	0.87	4.81	0.97	3.66	1.07	1.59	0.90	4.74	0.95
No CON No CHR	4.62	0.79	4.80	0.93	3.69	1.03	1.71	0.96	4.59	0.97
No CHR No THE	4.59	0.90	4.74	0.95	3.58	1.11	1.68	1.00	4.64	0.95
No CON No THE	4.50	0.89	4.73	0.94	3.67	1.06	1.70	0.94	4.64	0.88
No CON No CHR No THE	4.42	0.87	4.64	0.91	3.62	1.01	1.75	0.97	4.52	0.84

### Effects of total narrative coherence

In a first research aim, we wanted to investigate whether the earlier observed main effect of total narrative coherence on social responses was replicable. To examine whether fully coherent narratives (CON CHR THE) were more positively socially responded to than fully incoherent narratives (No CON No CHR No THE), an RM MANOVA was first conducted to examine if there was an effect of total narrative coherence on social responses in general (sum score of the different dependent variables, reverse scored for negative attitude).

For positive narratives, the RM MANOVA indicated a significant effect of total narrative coherence (NC) on the general social response level, *F*(1, 106) = 29.34, *p* < .001, *η*_*p*_^*2*^ = .22, *M*_*NC*_ = 4.62, *SE*_*NC*_ = 0.06, *M*_*NoNC*_ = 4.29, *SE*_*NoNC*_ = 0.07. For negative narratives, there was no significant effect of coherence on the general social response, *F*(1, 106) = 0.12, *p* = .74, *η*_*p*_^*2*^ = .001. RM ANOVAs were conducted for each dependent variable, both for positive and for negative narratives. As presented in Table 2, results show that there were strong beneficial effects of total narrative coherence on all different social responses, at least when narratives concerned positive topics. For negative narratives however, there were no effects of total coherence on any of the dependent variables. This is in line with our prediction that in particular for positive narratives, coherence is important to evoke positive social responses from listeners, whereas for negative narratives coherence does not account for significant differences in listeners’ reactions.

**Table 2:** Effects of total coherence of negative and positive autobiographical narratives on social responses

	F	p	η_p_^2^
*Negative*			
Willingness	< .001	.99	< .001
Emotional support	< .001	1.00	< .001
Instrumental support	.47	.49	.004
Negative attitude	.06	.81	.001
Positive attitude	.28	.60	.003
*Positive*			
Willingness	21.93	< .001	.17
Emotional support	20.97	< .001	.17
Instrumental support	12.12	.001	.10
Negative attitude	6.55	.012	.06
Positive attitude	31.16	< .001	.23

*Note*. *F*-tests with *df* (1,106)

### Effects of dimensions of coherence

Our second research aim concerned the investigation of the effects of the individual dimensions of coherence (context, chronology, theme) on listeners’ social responses. Since there was no effect of the total coherence of negative narratives on social responses (see Table 2), the following analyses were only run for positive narratives. The RM MANOVA comparing eight different levels of coherence in positive narratives on their general social reactions, was significant, *F(*7, 742) = 4.36, *p* < .001, *η*_*p*_^*2*^ = .04. The RM MANOVA in which context, chronology and theme were used as within-subject factors (2 × 2 × 2) showed that each individual dimension of coherence had a significant effect on the general social response: context, *F*(1, 106) = 4.74, *p* = .03, *η*_*p*_^*2*^ = .04, *M*_*CON*_ = 4.67, *SE*_*CON*_ = 0.06, *M*_*NoCON*_ = 4.39, *SE*_*NoCON*_ = 0.06; chronology, *F*(1, 106) = 14.53, *p* < .001, *η*_*p*_^*2*^ = .12, *M*_*CHR*_ = 4.48, *SE*_*CHR*_ = 0.06, *M*_*NoCHR*_ = 4.37, *SE*_*NoCHR*_ = 0.06 and theme, *F*(1, 106) = 10.70, *p* = .001, *η*_*p*_^*2*^ = .09, *M*_*THE*_ = 4.48, *SE*_*THE*_ = 0.06, *M*_*NoTHE*_ = 4.33, *SE*_*NoTHE*_ = 0.06. Below, results of 2 × 2 × 2 RM ANOVAs are described for each of the separate dependent variables, in order to investigate what the individual dimensions’ effects on different types of social reactions are.

### Willingness to interact

**Fig. 1:**
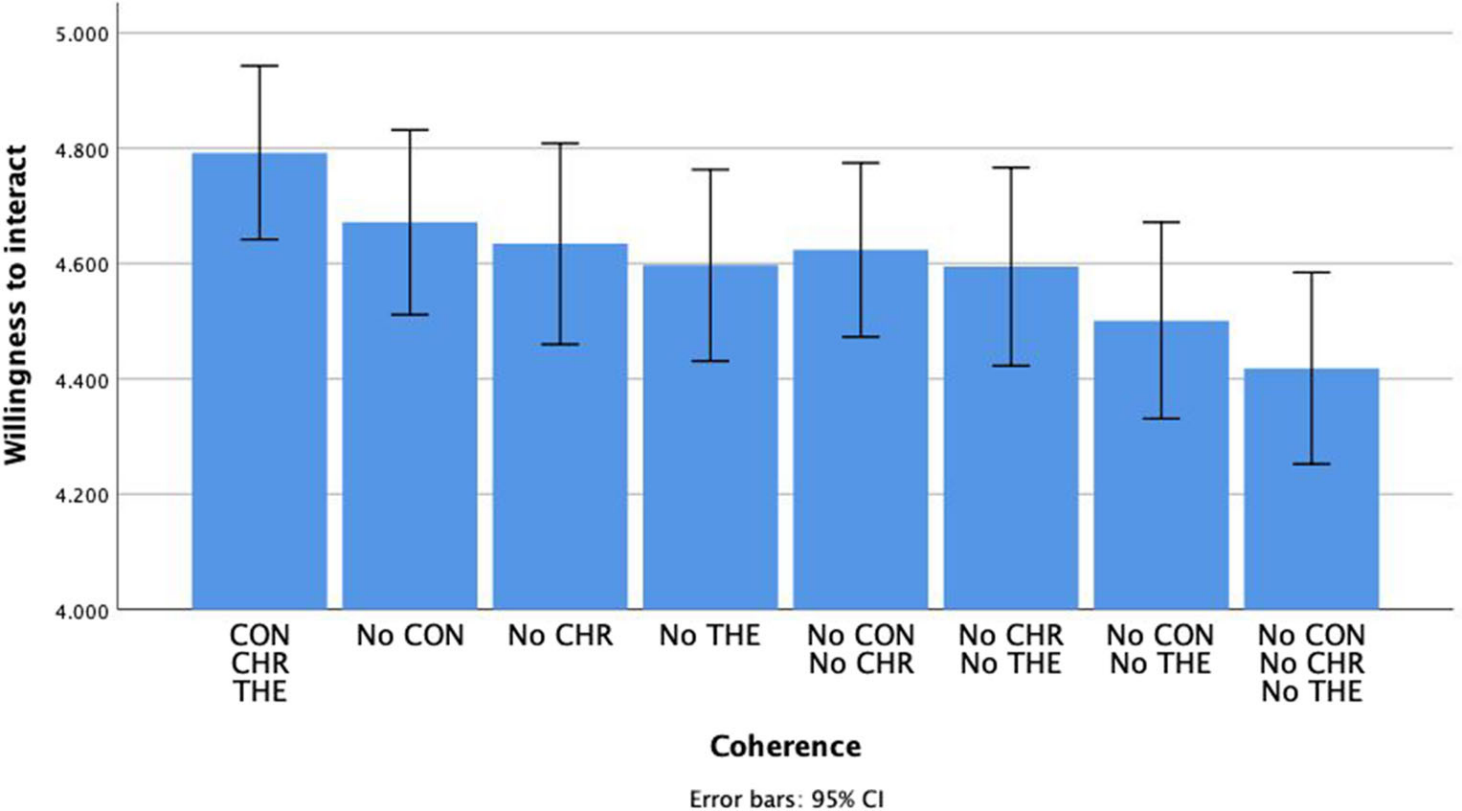
The impact of the coherence of positive autobiographical narratives on willingness to interact

All three individual dimensions had a significant effect on willingness to interact: context, *F*(1, 106) = 4.52, *p* = .04, *η*_*p*_^*2*^ = .04, *M*_*CON*_ = 4.65, *SE*_*CON*_ = 0.07, *M*_*NoCON*_ = 4.55, *SE*_*NoCON*_ = 0.07; chronology, *F*(1, 106) = 4.91, *p* = .03, *η*_*p*_^*2*^ = .04, *M*_*CHR*_ = 4.64, *SE*_*CHR*_ = 0.07, *M*_*NoCHR*_ = 4.57, *SE*_*NoCHR*_ = 0.07 and theme, *F*(1, 106) = 15.88, *p* < .001, *η*_*p*_^*2*^ = .13, *M*_*THE*_ = 4.68, *SE*_*THE*_ = 0.07, *M*_*NoTHE*_ = 4.53, *SE*_*NoTHE*_ = 0.07. The effect of theme was the largest, compared to the effects of context and chronology, which were of similar magnitude. None of the interaction effects between different dimensions were significant. See Fig. 1

### Emotional support

**Fig. 2:**
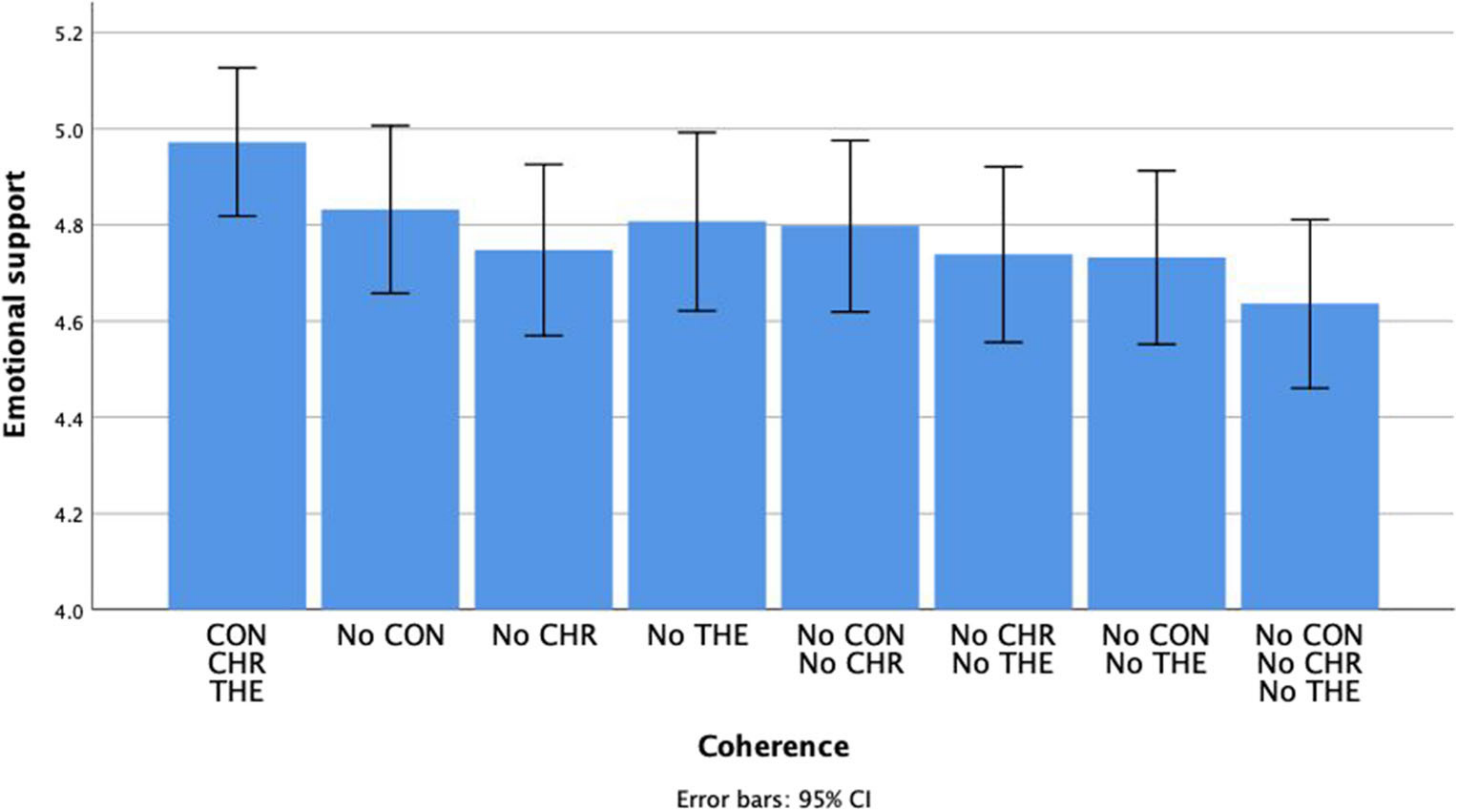
The impact of the coherence of positive autobiographical narratives on emotional support

For emotional support, only the dimension of chronology, *F*(1, 106) = 9.00, *p* = .003, *η*_*p*_^*2*^ = .08, *M*_*CHR*_ = 4.84, *SE*_*CHR*_ = 0.07, *M*_*NoCHR*_ = 4.73, *SE*_*NoCHR*_ = 0.08 and theme, *F*(1, 106) = 5.99, *p* = .02, *η*_*p*_^*2*^ = .05, *M*_*THE*_ = 4.84, *SE*_*THE*_ = 0.07, *M*_*NoTHE*_ = 4.73, *SE*_*NoTHE*_ = 0.08, had a significant effect. There was no main effect of context, *F*(1, 106) = 3.22, *p* = .08, *η*_*p*_^*2*^ = .03, neither were there any interaction effects between different dimensions. See Fig. 2

### Instrumental support

**Fig. 3:**
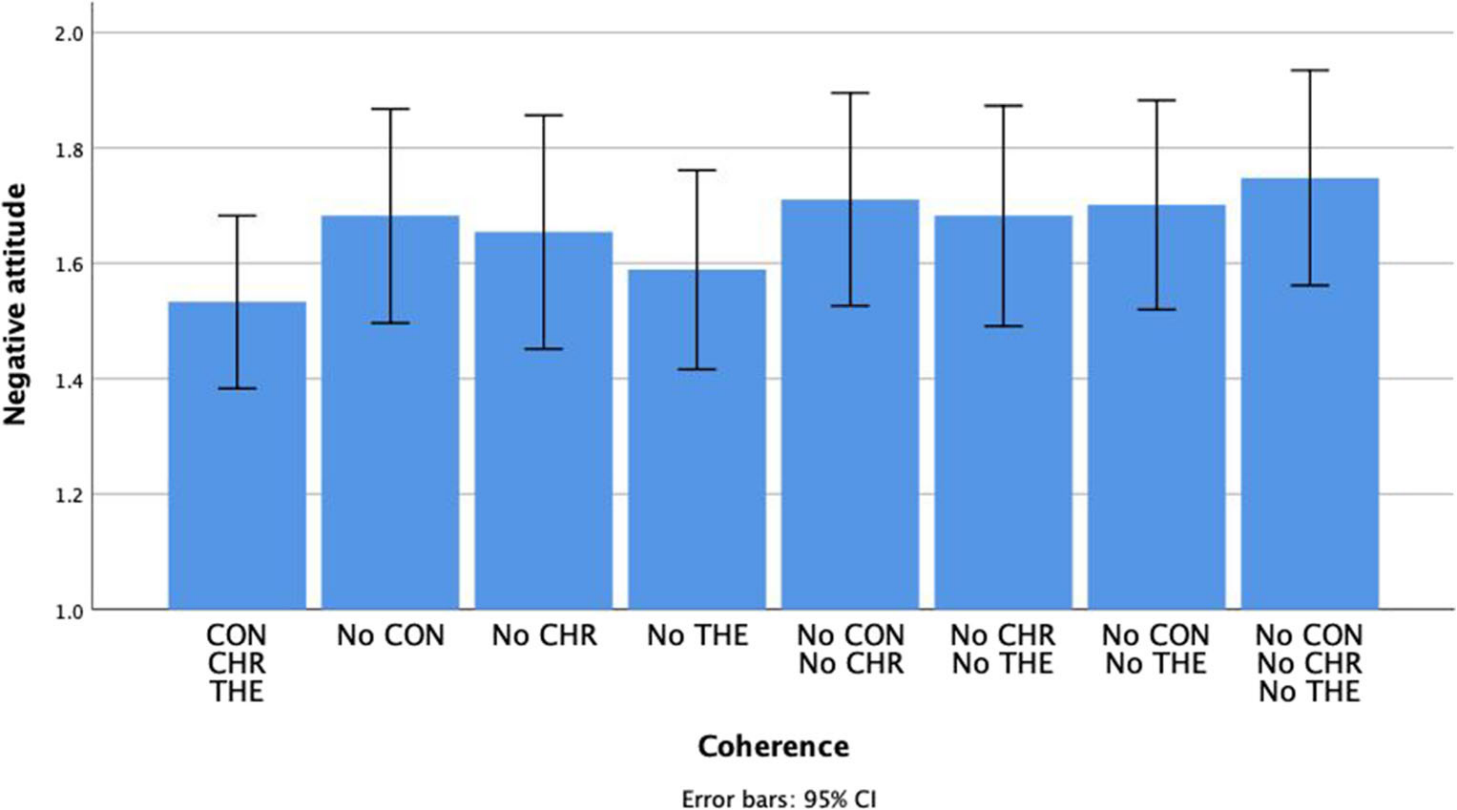
The impact of the coherence of positive autobiographical narratives on instrumental support

Results for instrumental support were similar compared to the results observed for emotional support. Only the dimension of chronology, *F*(1, 106) = 8.03, *p* = .005, *η*_*p*_^*2*^ = .07, *M*_*CHR*_ = 3.74, *SE*_*CHR*_ = 0.09, *M*_*NoCHR*_ = 3.64, *SE*_*NoCHR*_ = 0.09 and theme, *F*(1, 106) = 6.21, *p* = .01, *η*_*p*_^*2*^ = .06, *M*_*THE*_ = 3.74, *SE*_*THE*_ = 0.09, *M*_*NoTHE*_ = 3.63, *SE*_*NoTHE*_ = 0.09, had a significant effect. There was no main effect of context, *F*(1, 106) = 0.002, *p* = .97, *η*_*p*_^*2*^ < .001, neither were there any interaction effects between different dimensions. See Fig. 3

### Negative attitude

**Fig. 4:**
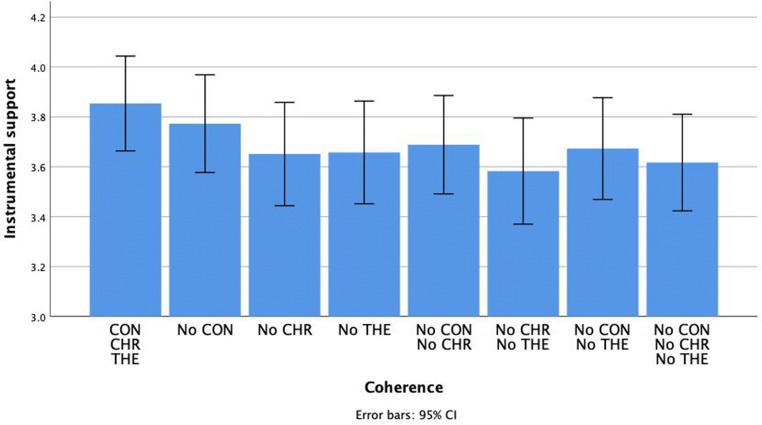
The impact of the coherence of positive autobiographical narratives on negative attitude

With regards to negative attitude, none of the dimensions of coherence had a significant effect: context, *F*(1, 106) = 3.67, *p* = .06, *η*_*p*_^*2*^ = .03, chronology, *F*(1, 106) = 2.33, *p* = .13, *η*_*p*_^*2*^ = .02, and theme, *F*(1, 106) = 0.61, *p* = .44, *η*_*p*_^*2*^ = .006, neither were there any interaction effects between dimensions. See Fig. 4.

### Positive attitude

For positive attitude, results were comparable to those of willingness to interact.

**Fig. 5:**
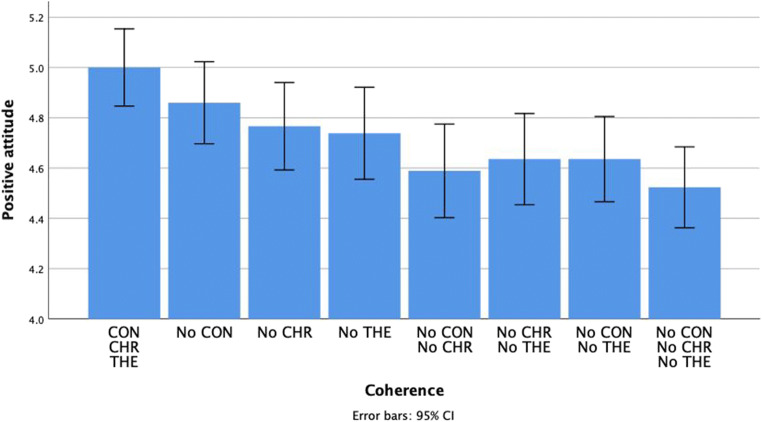
The impact of the coherence of positive autobiographical narratives on positive attitude

All three individual dimensions had a significant effect on positive attitude: context, *F*(1, 106) = 7.44, *p* = .007, *η*_*p*_^*2*^ = .07, *M*_*CON*_ = 4.79, *SE*_*CON*_ = 0.07, *M*_*NoCON*_ = 4.65, *SE*_*NoCON*_ = 0.07; chronology, *F*(1, 106) = 16.34, *p* < .001, *η*_*p*_^*2*^ = .13, *M*_*CHR*_ = 4.81, *SE*_*CHR*_ = 0.06, *M*_*NoCHR*_ = 4.63, *SE*_*NoCHR*_ = 0.07 and theme, *F*(1, 106) = 12.78, *p* = .001, *η*_*p*_^*2*^ = .11, *M*_*THE*_ = 4.80, *SE*_*THE*_ = 0.06, *M*_*NoTHE*_ = 4.60, *SE*_*NoTHE*_ = 0.07. Again, no interaction effects were observed. See Fig. 5.

### Multidimensional impact on general social scores

For positive stories, we did further analyses, additional to the pre-registered ones, to investigate the effects of leaving out one or more dimension(s) on social responses in general. Per dependent variable, narratives were put in the order from being negatively socially evaluated (less willingness, less emotional support, less instrumental support, more negative and less positive attitude) to being positively socially evaluated (more willingness, more emotional support, more instrumental support, less negative and more positive attitude) and respectively were assigned a score from 1 till 8. Then, across all five dependent variables, the scores were summed up per narrative. Using this method, narratives that were negatively socially evaluated received a low score, whereas more positively evaluated ones received a high score.

In Table 3, the social scores for all positive narratives are presented. Clearly, across all outcome variables, the completely coherent positive narratives were evaluated most positively and the completely incoherent ones most negatively, rendering respectively the highest and lowest score on all social outcomes.

**Table 3:** The multidimensional impact of the coherence of positive autobiographical narratives on total social scores

	Social score
CON CHR THE	40
No CON	33
No CHR	25
No THE	26
No CON No CHR	20
No CHR No THE	15
No CON No THE	15
No CON No CHR No THE	6

When leaving out one dimension, the biggest detrimental effect on social responses was caused by the dimension of theme, closely followed by leaving out the dimension of chronology. Leaving out context was not crucial in impacting social responses, and was situated somewhere in between the completely coherent narratives, and the ones with missing chronology or theme.

When two dimensions lacked in the narrative, responses were more negative than when only one dimension was lacking. When one of the two dimensions concerned theme, irrespective of the other missing dimension (chronology or context), the detrimental effect on social responses was bigger than when theme was still present. In that case, when theme was present, but context and chronology absent, responses were still worse than when only one dimension lacked in the narrative, but higher than when theme was absent as one of the two missing dimensions. In general, we can conclude that, for narratives about a high point, narrative coherence does have a positive impact on social responses. The higher the coherence, the higher listeners think of the narrator. The more elements are missing in terms of coherence, the worse the social response becomes. When the narrative is lacking in coherence, it seemed that the absence of chronological and mainly thematic coherence was crucial in deteriorating the social response.

## Discussion

The aims of this experimental study were twofold. First, we wanted to investigate whether the positive effect of narrative coherence on listeners’ social responses, observed in Vanaken et al. (2020), was replicable. Second, we wanted to extend and refine previous findings by examining the multidimensional impact of coherence in positive and negative narratives on listeners’ social responses.

In line with our predictions, we could replicate the effect of coherence on listeners’ social responses that was described in Vanaken et al. (2020). In this study, listeners showed more willingness to interact, more emotional support, more instrumental support, a more positive and a less negative attitude towards those that narrated upon their autobiographical memory in a fully coherent manner (CON = 3, CHR = 3, THE = 3), in comparison to towards those that did so fully incoherently (CON = 0, CHR = 0, THE = 0). However, whereas in Vanaken et al. (2020), narrative coherence generally had an overruling effect, regardless of the valence of the narrative, in the current study, the effects of coherence were only present in narratives about positive autobiographical experiences. Nonetheless, the current findings are in line with the interaction effect observed in Vanaken et al. (2020), showing that negative feelings in the listener were evoked when narratives were incoherent, but only if the narrative concerned a positive topic, and with previous literature showing that positive memories are more adept at serving social functions (Alea et al., 2013; McLean & Lilgendahl, 2008; Rasmussen & Berntsen, 2009). In our study, incoherence in negative narratives was not negatively socially responded to, possibly because incoherence in negative stories could be seen as an integral part to an ongoing meaning-making process. Indeed the research of Bisby, Horner, Bush and Burgess (2018) lends support to the idea that negative emotional content, and especially traumatic content, can disturb the coherence of autobiographical memories. Furthermore, listeners could be more tolerant or more habituated towards incoherent negative stories, since the help of loved ones is often sought after going through a low point, for compassion reasons (Duprez, Christophe, Rimé, Congard, & Antoine, 2015) or in order to co-construct a logically ordered and emotionally regulated narrative (Fivush & Sales, 2006; Pasupathi, 2001). Note, however, that these post hoc explanations need to be interpreted with caution and require further investigation.

Our second research aim concerned identifying the crucial dimensions that impact social responses. We found that, generally, all three dimensions of narrative coherence had an effect on listeners’ social reaction. However, when looking at the specific social responses separately, chronology and theme seemed to be the most important, since they had a significant effect on all social outcomes. Context only affected willingness and positive attitude but did not impact emotional and instrumental support. Remarkably, with regard to one social outcome, negative attitude, none of the individual dimensions of coherence had a significant effect. Possibly, participants are more hesitant to openly showing a negative attitude towards someone when participating in research, because of social desirability. The other social responses included in this study are more neutrally or positively framed, which could possibly allow for more honesty and hence variability in answers. Besides negative attitude, the results on social outcomes were largely in line with our second prediction that particularly the absence of chronology or theme would be negatively socially responded to, whereas a missing context would not be significantly negatively reacted upon.

These results can be explained by two possible mechanisms. The first concerns the reduction of the attraction effect in cases of increased effortful processing (Tsuzuki, Takeda, & Chiba, 2019). In particular, we suggest that chronological incoherence might set in motion increased effortful processing. According to the dual processing model of cognition, the evaluation of other people occurs in two consecutive stages (Brewer, 1988). The first is a rather automatic top-down driven stage, whereas the second is a more controlled phase, relying on bottom-up information processing (Brewer, 1988). Consequently, the switch from the first to the second stage requires increased cognitive resources, also referred to as attentional effort or effortful processing. Furthermore, social cognition is not independent from social affect (Wyer & Srull, 1986). Relatedly, research has demonstrated that this increased allocation of cognitive resources reduces the initial attraction effect (Tsuzuki et al., 2019). The more attentional effort it takes to process information, the lower attraction will be. Therefore, it might be possible that a chronologically incoherent story is difficult to follow for the listener, requiring a great deal of cognitive resources, which might result in reduced attraction towards the narrator, expressed in lower scores on positive social measures like willingness to interact, social support, and positive attitude.

The second candidate mechanism concerns the idea that coherence might be a necessary condition to establish credibility in a story (Elleström, 2018). Coherence is throughout the narrative literature described as the ultimately necessary condition for a high-quality narrative (Reese et al., 2011), and as the fundamental property of a story (Adler, Wagner, & McAdams, 2007). Relatedly, semiotic psychology has evidenced the idea that coherence is the basis of establishing credibility in communication (Elleström, 2018). Conway has worked on this idea as well, suggesting that memories that score low on internal coherence and low on external correspondence are often categorized by outsiders as confabulated memories (Conway, 2005; Conway, Singer, & Tagini, 2004). Applied to our findings, it might be the case that incoherence gives listeners the impression of a less credible narrator, increasing distrust and decreasing the willingness for future interactions or social support. Specifically, when narratives score low on thematic coherence, emotional elaborations and personal interpretations are lacking, possibly giving the listener the impression that the narrator is making up the story, as if he/she did not in fact experience the narrated event him-/herself. Therefore, in particular thematic incoherence might establish distrust in a relationship, expressed in this study in lower scores on our investigated social measures. Of course, the two suggested mechanisms above are not mutually exclusive, neither do they rule out other possible mediators. Future experimental research is recommended to investigate these and other suggested mechanisms.

Additionally, we investigated the multidimensional impact of coherence of positive narratives across all social responses. As expected, we observed an additive effect of the dimensions of coherence; the more dimensions were missing in the narrative, the more negative the social response. When one dimension was missing, the absence of chronology and theme had comparable detrimental effects, whereas a missing context was not reacted upon in a significantly worse way. Furthermore, when two dimensions were missing, social scores were especially lower when one of those two dimensions concerned theme. Overall, lowest scores were given to narratives missing all three dimensions.

The present results add significant value to the domain of memory and cognition. This study in fact establishes the idea that cognitive psychological research would benefit from extending the intra-individual perspective to include an inter-individual perspective that considers social aspects of memory and cognition as well. As addressed earlier, social responses of others to our memory-sharing behavior can impact our psychological well-being, given the universal need of humans to experience a sense of belongingness (Baumeister & Leary, 1995). When social support is limited or absent, risks for mental and physical health problems are severely heightened (Harandi et al., 2017; Ozbay et al., 2007). Up to now, research has mainly focused on the importance of social support in the face of negative life events or trauma resilience (Charney, 2004; Ozer, Best, Lipsey, & Weiss, 2003; Sippel, Pietrzak, Charney, Mayes, & Southwick, 2015; Southwick, Bonanno, Masten, Panter-Brick, & Yehuda, 2014). Importantly, the innovativeness of our data lies in the idea that social support is not only crucial after experiencing a negative or traumatic event, but at least as much so after experiencing a positive event. Not only do people share positive events very often (Rimé et al., 1998), positive events specifically, and more so than negative events, are adept at increasing positive emotion and building that necessary supportive network that can become a protective factor for mental health in case future adversities arise (Duprez et al., 2015, McLean & Lilgendahl, 2008).

In fact, reasons as to why people especially express positive memories to others are suggested to be twofold – capitalization and social integration. Capitalization is a term originating from Langston (1994), and refers to the enhancement of positive affect far beyond the actual positive effect of the event, when communicating about it to other people. In other words, sharing a positive event after experiencing it causes individuals to feel significantly more positive emotion than is actually attributable to the event itself. However, when doing so in an incoherent manner, it is likely that the additional beneficial effects of positive narration are limited. Especially when thematic coherence is low, emotional expression of the experience is confined, creating less personal reliving and less enhancement of positive affect through narration. Furthermore, the positive response of others towards such capitalization of the subject is thought to increase feelings of interpersonal closeness and intimacy, thereby enhancing social integration and relationship satisfaction (Gable, Reis, Impett, & Asher, 2004; Reis et al., 2010). However, when narration is incoherent, positive social reinforcement will decline, reducing feelings of belonging to a supportive social network, and increasing feelings of loneliness and depression, hence compromising mental health (Baumeister & Leary, 1995; Harandi et al., 2017). Therefore, the incoherent narration of positive experiences in particular might be detrimental to the subject’s well-being, via direct (no capitalization) as well as indirect (no social integration) pathways. Since these pathways are suggested to develop over time, when incoherent narration endures and a negative social interaction spiral becomes gradually worse (Coyne, 1976), future research could longitudinally investigate how incoherent narrators experience their social and psychological well-being evolving over time.

### Limitations

Certain limitations can be addressed in the current study, which mainly concern the experimental nature of the design. Carrying the advantage of having a higher internal validity, experiments do have the downfall of being lower in external, or ecological validity. We took an in-depth approach to investigate merely, but in a very refined manner, the multidimensional impact of coherence in narratives. Naturally, a large number of other variables that are thought to play a role in narratives and their relation to social or psychological outcomes were ruled out in this study, with the purpose of having a clear measure of the effect under interest, without possible interference of third variables. For instance, in memory and narrative research, inclusion of different subjective perspectives, the specificity of the described event, the similarity of the described event type to the listener’s life, etc., are all variables that have shown to be important, but were ruled out in this experimental design (Habermas, 2019; Habermas & Diel, 2010; Williams et al., 2007). However, the multifaceted nature of narratives remains a challenge. Whilst adhering to a strictly controlled experimental design, which entails counterbalancing and randomizing narratives across participants, we cannot completely rule out the possible effect of other confounding variables (e.g., self-disclosure, social content). Therefore, it would be very interesting to consider other narrative variables in a follow-up study, since we currently do not know of any existing evidence regarding the covariance of those variables with our measure of narrative coherence.

Furthermore, a range of social-contextual variables as well as cultural differences, like the type and length of the relationship, listeners’ responsiveness, narrator-listener familiarity and similarity (e.g., gender), individualistic versus collectivistic nature of the culture, etc., were also all kept constant, although recognized as valuable research topics (Alea & Bluck, 2003, Bavelas et al., 2000; Fivush, Bohanek, Zaman, & Grapin, 2012; Fivush & Nelson 2004; Grysman, Fivush, Merrill, & Graci, 2016; Reese, Haden, & Fivush, 1996). We deliberately chose to work, for both narrators and listeners, with a defined group of predominantly female, white, highly educated, young adults, since individual differences in emotional reactions are more outspoken in dyads that are highly similar (Alea & Bluck, 2003). Of course, the downside of having a concise and homogeneous group of participants limits the generalizability of the results with regard to gender and culture. Future research could adopt a more inclusive approach to investigate these possible differences.

### Conclusion

In our experimental study, we replicated previously observed findings (Vanaken et al., 2020), indicating that narrative coherence has important beneficial effects on listeners’ social responses. More specifically, listeners showed more willingness to interact, more emotional support, more instrumental support, a more positive attitude, and a less negative attitude towards those who narrated in a coherent manner, as opposed to those who narrated in an incoherent manner. Remarkably, in this study, the described effects of coherence were only observed in narratives about positive events. Results were explained in the light of the importance of positive memories for the social bonding function of autobiographical memory. Furthermore, we observed in line with our predictions that the dimensions of chronology and, predominantly, theme were most important in impacting social responses of listeners. When narratives missed chronological or thematic coherence, responses were significantly worse compared to narratives that were fully coherent or narratives missing context. Possibly a reduction of the attraction effect due to increased effortful processing, and reduced credibility due to insufficient emotional elaboration might explain these results, respectively. When narratives missed two dimensions social scores were lower than when narratives lacked in only one dimension, in line with the expected additive effect. Effects were even more outspoken particularly when one of those two missing dimensions concerned theme. Overall, fully incoherent narratives, meaning low scores on context, chronology, and theme, had the most adverse effect on listeners’ social responses. The importance of coherently sharing positive memories for the field of memory and cognition is addressed by highlighting the value of broadening the focus from a merely intrapersonal perspective to include an interpersonal view on memory processes as well. The limitations of the present research have been discussed and future research ideas formulated.

## Appendix 1: Exemplary narratives

Below, we provide eight versions of one narrative which concerned a positive topic. The eight versions systematically differ in terms of narrative coherence (Reese et al., 2011). All 128 narratives that were used in the study are provided on the OSF page of the study (10.17605/OSF.IO/3CTXV ). Note that these narratives are translated from Dutch, so English translations may slightly differ from the original Dutch text.

### Positive topic: falling in love

#### CON CHR THE

A memory that stands out for me, is meeting my boyfriend. We went to high school together but didn’t really know each other back then. However, when we went on a school trip to Rome in our last year of high school, that completely changed. By accident, I ended up next to him on the bus, and we started talking from that moment on. I immediately noticed how comfortable I felt talking to him and sitting next to him. During that whole school trip, we talked a lot, laughed a lot, and had an amazing time together. I can definitely say that that was one of the best journeys ever! Once we were back in Belgium, we met up on the weekend and went for a walk in the forest, nearby my parents’ house. We were about halfway on our walk, quite far into the woods. There was no one around us, when suddenly, he took my hand. We looked into each other’s eyes and that eye contact was very intense. Then, he put his hand behind my head and very carefully kissed me. That moment felt so special! I was completely smitten and felt so happy. I didn’t want to let go of him, everything felt so right. I often still think about that moment and realize how grateful I am that I have met someone who is so caring, who fits me so well. I wouldn’t want to miss him for the world. We do a lot of fun things together and just make the best memories. Also, still now, after being together for 5 years, I love him more every day.

#### No CON

A memory that stands out for me, is meeting my boyfriend. We grew up together but didn’t really know each other back then. However, when we went on a school trip, that completely changed. By accident, I ended up next to him on the way there, and we started talking from that moment on. I immediately noticed how comfortable I felt talking to him and sitting next to him. During that whole school trip, we talked a lot, laughed a lot, and had an amazing time together. I can definitely say that that was one of the best journeys ever!

Once we were back, we met up on the weekend and went for a walk. When we were about halfway on our walk, and no one was around, he suddenly took my hand. We looked into each other’s eyes and that eye contact was very intense. Then, he put his hand behind my head and very carefully kissed me. That moment felt so special! I was completely smitten and felt so happy. I didn’t want to let go of him, everything felt so right. I often still think about that moment and realize how grateful I am that I have met someone who is so caring, who fits me so well. I wouldn’t want to miss him for the world. We do a lot of fun things together and just make the best memories. Also, still now, after being together for so long, I love him more every day.

#### No CHR

That moment felt so special! That whole trip, we talked and laughed a lot and just had a really good time. I was completely smitten and so happy. I noticed immediately how comfortable I felt talking to him and sitting next to him. Back in Belgium, we met up on the weekend and went for a walk in the forest, nearby my parents’ house. We went to high school together but didn’t really know each other yet. We were about halfway, far into the forest. No one was around, and he took my hand. I often think about that moment and realize how grateful I am that I have met someone who is so caring, who fits me so well.

We looked into each other’s eyes and that eye contact was very intense. He put his hand behind my head and very carefully kissed me. Actually, everything changed during that school trip to Rome, which was in the last year of high school. By accident, I ended up next to him on the bus, and we started talking. I wouldn’t want to miss him for the world. After being together for 5 years, I love him more and more every day. I can definitely say that that was one of the best journeys ever! I didn’t want to let go of him, everything just felt right. We do so many fun things and make the best memories together.

#### No THE

One of my memories is meeting my boyfriend. We went to high school together but didn’t really know each other back then. However, when we went on a school trip to Rome in our last year of high school, that completely changed. By accident, I ended up next to him on the bus, and we started talking from that moment on. The conversation ran smoothly. That whole trip we talked a lot. Once we were back in Belgium, we met up on the weekend and went for a walk in the forest, nearby my parents’ house. We were about halfway on our walk, quite far into the woods. There was no one around us, when suddenly, he took my hand. We looked into each other’s eyes. Then, he put his hand behind my head and kissed me. I didn’t want to let go of him. I can remember that moment. We do a lot of things together still to this day, make a lot of memories, even after being together for 5 years already.

#### No CON No CHR

That moment felt so special! That whole trip, we talked and laughed a lot and just had a really good time. I was completely smitten and so happy. I noticed immediately how comfortable I felt talking to him and sitting next to him. Once we were back, we met up on the weekend and went for a walk. We grew up in the same area but didn’t really know each other yet. We were about halfway on our walk, no one was around, and he took my hand. I often think about that moment and realize how grateful I am that I have met someone who is so caring, who fits me so well. We looked into each other’s eyes and that eye contact was very intense. He put his hand behind my head and very carefully kissed me. Actually, everything changed during that school trip. By accident, I ended up next to him on the way there, and we started talking. I wouldn’t want to miss him for the world. After being together for so long, I love him more and more every day. I can definitely say that that was one of the best journeys ever! I didn’t want to let go of him, everything just felt right. We do so many fun things and make the best memories together.

#### No CHR No THE

That moment, wow! That whole trip, we talked a lot. The conversation ran smoothly. Back in Belgium, we met up on the weekend and went for a walk in the forest, nearby my parent’s house. We went to high school together but didn’t really know each other back then. We were about halfway on our walk, far into the forest. No one was around, and he took my hand. We looked deep into each other’s eyes. He put his hand behind my head and kissed me. Actually, everything thus changed in the last year of high school, during that school trip to Rome. By accident, I ended up next to him on the bus, and we started talking. I didn’t want to let go of him. I still often think about that moment. We do a lot of things together and make memories, also still now, after being together for 5 years.

#### No CON No THE

One of my memories is meeting my boyfriend. We grew up in the same area but didn’t really know each other back then. However, when we went on a school trip together, that completely changed. By accident, I ended up next to him on the way there and we started talking. The conversation ran smoothly right away. That whole trip we talked a lot. Once we were back, we met up on the weekend and went for a walk. We were about halfway on our walk, and no one was around, when he suddenly took my hand. We looked into each other’s eyes. Then, he put his hand behind my head and kissed me. I didn’t want to let go of him. I still think about that moment often. We do a lot of things together still to this day, make a lot of memories, even after being together for so long.

#### No CON No CHR No THE

That moment, wow! That whole trip, we talked a lot. The conversation ran smoothly. Once we were back, we met up on the weekend and went for a walk. We grew up in the same area but didn’t really know each other back then. We were about halfway on our walk, far into the forest. No one was around, and he took my hand. We looked deep into each other’s eyes. He put his hand behind my head and kissed me. Actually, everything changed on that school trip. By accident, I ended up next to him on the way there, and we started talking. I didn’t want to let go of him. I still often think about that moment. We do a lot of things together and make memories, also still now, after being together for so long.
